# Hypomyelination caused by a novel homozygous pathogenic variant in *FOLR1*: complete clinical and radiological recovery with oral folinic acid therapy and review of the literature

**DOI:** 10.1186/s13023-023-02802-6

**Published:** 2023-07-13

**Authors:** Ana Potic, Stefanie Perrier, Tijana Radovic, Svetlana Gavrilovic, Jelena Ostojic, Luan T. Tran, Isabelle Thiffault, Tomi Pastinen, Raphael Schiffmann, Geneviève Bernard

**Affiliations:** 1 Clinic for Child Neurology and Psychiatry, Department of Neurology, University of Belgrade, 6A Dr. Subotica Street, 11000 Belgrade, Serbia; 2 Departments of Neurology and Neurosurgery, McGill University, Montreal, Canada; 3 Child Health and Human Development Program, Research Institute of the McGill University Health Centre, Montreal, Canada; 4 University Children’s Hospital, Department of Radiology, University of Belgrade, Belgrade, Serbia; 5 University Clinical Centre of Serbia, Centre for Radiology and Magnetic Resonance, University of Belgrade, Belgrade, Serbia; 6 Faculty of Medicine, Department of Radiology, University of Novi Sad, Novi Sad, Serbia; 7 Genomic Medicine Center, Children’s Mercy Hospital, Kansas City, MO USA; 8 University of Missouri Kansas City School of Medicine, Kansas City, MO USA; 9 Department of Pathology and Laboratory Medicine, Children’s Mercy Hospital, Kansas City, MO USA; 10 Texas Neurology, 6080 N Central Expy Ste 100, Dallas, TX USA; 11 Departments of Pediatrics and Human Genetics, McGill University, Montreal, Canada; 12 Department Specialized Medicine, Division of Medical Genetics, McGill University Health Centre, Montreal, Canada

**Keywords:** *FOLR1*, Hypomyelination, Leukodystrophy, Cerebral folate deficiency, Folinic acid

## Abstract

**Background:**

Neurodegeneration due to cerebral folate transport deficiency is a rare autosomal recessive disorder caused by biallelic pathogenic variants in *FOLR1*. Onset typically occurs in late infancy and is characterized by psychomotor regression, epilepsy, and a hypomyelinating leukodystrophy on magnetic resonance imaging. If left untreated, progressive neurodegeneration occurs. However, early treatment with folinic acid has been shown to stabilize or reverse neurological features. Approximately thirty patients have been described worldwide. Here, we report the first two cases with genetically proven cerebral folate transport deficiency from South-Eastern Europe, describe the effect of oral folinic acid therapy on clinical and neuroradiological features and review the literature.

**Results:**

Two siblings presented in childhood with clinical and radiological findings consistent with a hypomyelinating leukodystrophy. Exome sequencing revealed a novel homozygous pathogenic variant in *FOLR1* (c.465_466delinsTG; p.W156G), confirming the diagnosis of neurodegeneration due to cerebral folate transport deficiency. Folinic acid treatment was promptly initiated in both patients. The younger sibling was treated early in disease course at 2 years of age, and demonstrated complete recovery in clinical and MRI features. The older sibling, who was 8 years of age at the time of diagnosis and treatment, demonstrated partial but substantial improvements.

**Conclusion:**

We present the first account in the literature that early treatment initiation with oral folinic acid alone can result in complete neurological recovery of both clinical and radiological abnormalities in neurodegeneration due to cerebral folate deficiency. Moreover, through the report of these patients along with review of the literature, we provide information about the natural history of the disease with comparison of treatment effects at different stages of disease progression. This report also reinforces the importance of universal access to genetic testing to ensure prompt diagnoses for treatable disorders.

## Background

Neurodegeneration due to cerebral folate transport deficiency (OMIM #613068), first described in 2009, is caused by biallelic pathogenic variants in *FOLR1* [1]. *FOLR1* (OMIM *136430) encodes for the folate receptor-alpha (FOLRα), which is abundantly expressed in the choroid plexus and considered the main folate transporter of 5-methyltetrahydrofolate (MTHF) across the blood–brain barrier. FOLRα is the only transporter responsible for cerebral folate supply via exosome-mediated delivery of MTHF from the CSF to the brain parenchyma [1–3].

Biallelic hypomorphic pathogenic variants in *FOLR1* cause FOLRα deficiency, impairing cerebral folate transport and supply, leading to isolated cerebral folate deficiency and progressive neurodegeneration [1–3]. This disorder typically starts to manifest in late infancy with psychomotor regression, ataxia, and refractory epilepsy, with brain magnetic resonance imaging (MRI) demonstrating a hypomyelinating leukodystrophy [1, 2].

The late-infantile onset and absence of embryonic malformations in this disorder suggest preserved expression of folate receptor-beta (FOLRβ) in fetal choroid cells, which compensates for the lack of FOLRα function [1, 2, 4]. However, downregulation of FOLRβ expression is thought to occur in the human choroid plexus from 4 to 6 postnatal months onwards, which may explain the onset of the disease only in late infancy [1, 3, 4].

The pathophysiological mechanisms by which MTHF deficiency causes neurological disease are still under investigation. The prevailing hypothesis links the lack of MTHF to impaired myelin formation through cerebral methylation processes, which results in a deficiency of phosphatidylcholine, sphingomyelin, and other methylated membrane phospholipids crucial for myelin formation and stability [1]. Another recent hypothesis posits that folates are important for oligodendrocyte maturation, survival, and thus for the myelination during CNS development [5].

Here, we report siblings with hypomyelination and neurodegeneration for whom exome sequencing revealed a homozygous novel pathogenic variant in *FOLR1.* We also present an in-depth report before and during folinic acid treatment, with clinical and MRI evolution, as well as a review of the previously published cases.

## Methods

### Ethics approval and research consent

This research was approved by the Institutional Review Boards of Clinic for Child Neurology and Psychiatry University of Belgrade (IRB number 1-48/3-2016) and the McGill University Health Center and Montreal Children’s Hospital Research Ethic Boards (11-105-PED and 2019-4972), and conducted following the 1964 Declaration of Helsinki and its later amendments. Written informed consent was obtained from the patients’ parents/legal guardians.

### Genetic analysis

Exome sequencing was performed using genomic DNA extracted from whole blood following standard protocols. DNA was prepared using the TruSeq library prep and samples were enriched using the IDT xGenv2 exome research panel supplemented with custom mitochondrial probes and sequenced to a minimum of 7 Gb for a mean of 80 × average coverage or greater on an Illumina NovaSeq 6000 (2 × 150 paired end reads). Bidirectional sequences were assembled, aligned to reference gene sequences based on human genome build GRCh37/UCSC hg19, and analyzed using the custom-developed software RUNES and VIKING [6, 7]. Variants were filtered to 1% minor allele frequency and prioritized using the American College of Medical Genetics and Genomics (ACMG) guidelines [8], including phenotypic assessment with OMIM disease associations.

### Medical record and MRI review

We retrospectively reviewed medical records and evaluated MRI studies conducted serially over 10 years for Patient 1 and over 4 years for Patient 2.

Further, we assessed the data from all published patients with biallelic pathogenic variants in *FOLR1*, considering their clinical features, neuroimaging results, genetic findings, treatment regimen, and response to treatment. This was completed by reviewing all biomedical literature available in the PubMed Medline database between September 2009-December 2022, using the following MeSH terms: FOLR1 gene, cerebral folate deficiency, hypomyelination, leukodystrophy, folinic acid.

### Serum folate measurements

Serum folate levels were measured using Abbott Architect i4000Sr test equipment and Abbott Architect Folate reagent using the Chemiluminescent Microparticle Immunoassay (CMIA) method.

## Results

The patients described in this study are siblings, including a boy currently aged 12 years (Patient 1), and his younger sister currently aged 6 years (Patient 2). They were born to non-consanguineous unaffected parents of Serbian origin, with a family history negative for neurological disorders. Both patients were referred to our department for additional investigations at the ages of 8 years (Patient 1, for epileptic encephalopathy) and 2 years (Patient 2, for mild cerebellar features).

### Pre-treatment clinical findings

The older male sibling (Patient 1) had uneventful early psychomotor development until 18 months of age, when he gradually developed an ataxic gait and speech regression. At 4 years of age, he started having epileptic seizures, which were treatment-resistant, occurred daily, and of multiple different types (tonic, focal with impaired awareness, atonic, and tonic–clonic). Various combinations of ten standard antiepileptic drugs (AEDs) were tried without success. At the time, the patient was being treated in a department without resources for access to detailed metabolic investigations or genetic sequencing, resulting in his cause of illness remaining unknown. Cerebellar ataxia and hypotonia progressed, and at 6.5 years of age, he lost the ability to walk and sit without support, with poor head control. His expressive language consisted of up to five meaningful words and he showed autistic behavioral changes with outbursts of anger and poor social contact. No other abnormalities were found on physical examination. The severity of his seizures increased, frequently leading to status epilepticus. EEG showed diffuse disturbance in cerebral activity with slow background activity and multifocal epileptiform discharges. The patient’s neurological motor, cognitive, and language function progressively worsened, leading to dependency for all activities of daily living and severe neurological impairment at 7.5 years of age. His examination at the time was characterized by a complete loss of speech and social interactions, as well as significant cerebellar signs (i.e., truncal and limb ataxia), axial hypotonia, and mild pyramidal and bulbar signs.

The younger female sibling (Patient 2) had normal psychomotor development. At the age of 22 months, she started manifesting mild intention tremor in the upper limbs and mild truncal ataxia. She did not exhibit seizures or any other neurological abnormalities.

### Pre-treatment brain MRI

**Fig. 1:**
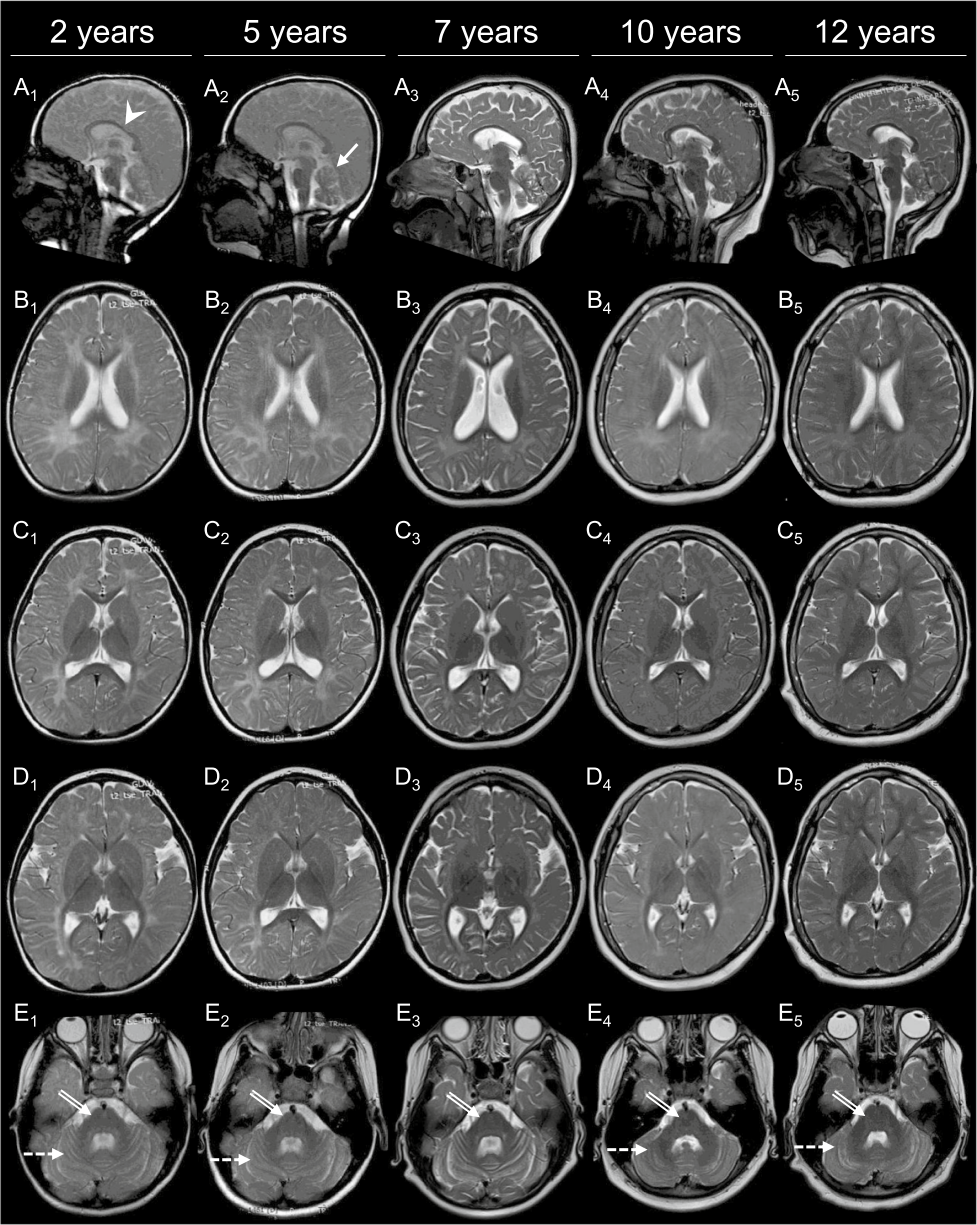
Brain MRI of Patient 1 from age 2 to 12 years. T2-weighted images are shown at 2 years (column 1: **A**_**1**_–**E**_**1**_), 5 years (column 2: **A**_**2**_–**E**_**2**_), 7 years (column 3: **A**_**3**_–**E**_**3**_), 10 years (column 4: **A**_**4**_–**E**_**4**_) and 12 years (column 5: **A**_**5**_–**E**_**5**_). Sagittal (panel **A**) images show mild to moderate thinning of the corpus callosum (white arrowhead), as well as mild cerebellar atrophy (white arrow). In panels **B**_**1–3**_, **C**_**1–3**_, and **D**_**1–3**_, severe lack of myelin deposition, together with progressive cerebral atrophy are appreciated. In panels **B**_**4–5**_, **C**_**4–5**_, and **D**_**4–5**_, improvement in myelination is seen, but incomplete myelination is still present at age 12 years. Of note, brain volume has improved at ages 10 and 12 years (**B**_4–5_, **C**_**4–5**_, and **D**_**4–5**_). In panel **E**, insufficient myelin deposition is seen in **E**_**1-2**_ in both the pons (white double-lined arrow) and cerebellum (white dashed arrow), with improvement in the pons at age 7 years (**E**_**3–5**_, white double-lined arrow) and significant improvement in the cerebellum at ages 10 and 12 years (**E**_**4–5**_, white double-lined arrows). Progressive cerebellar atrophy is also seen between ages 2 and 7 years (**E**_**1–3**_), with improvement in subsequent MRIs done at ages 10 and 12 years (**E**_**4–5**_)

**Fig. 2:**
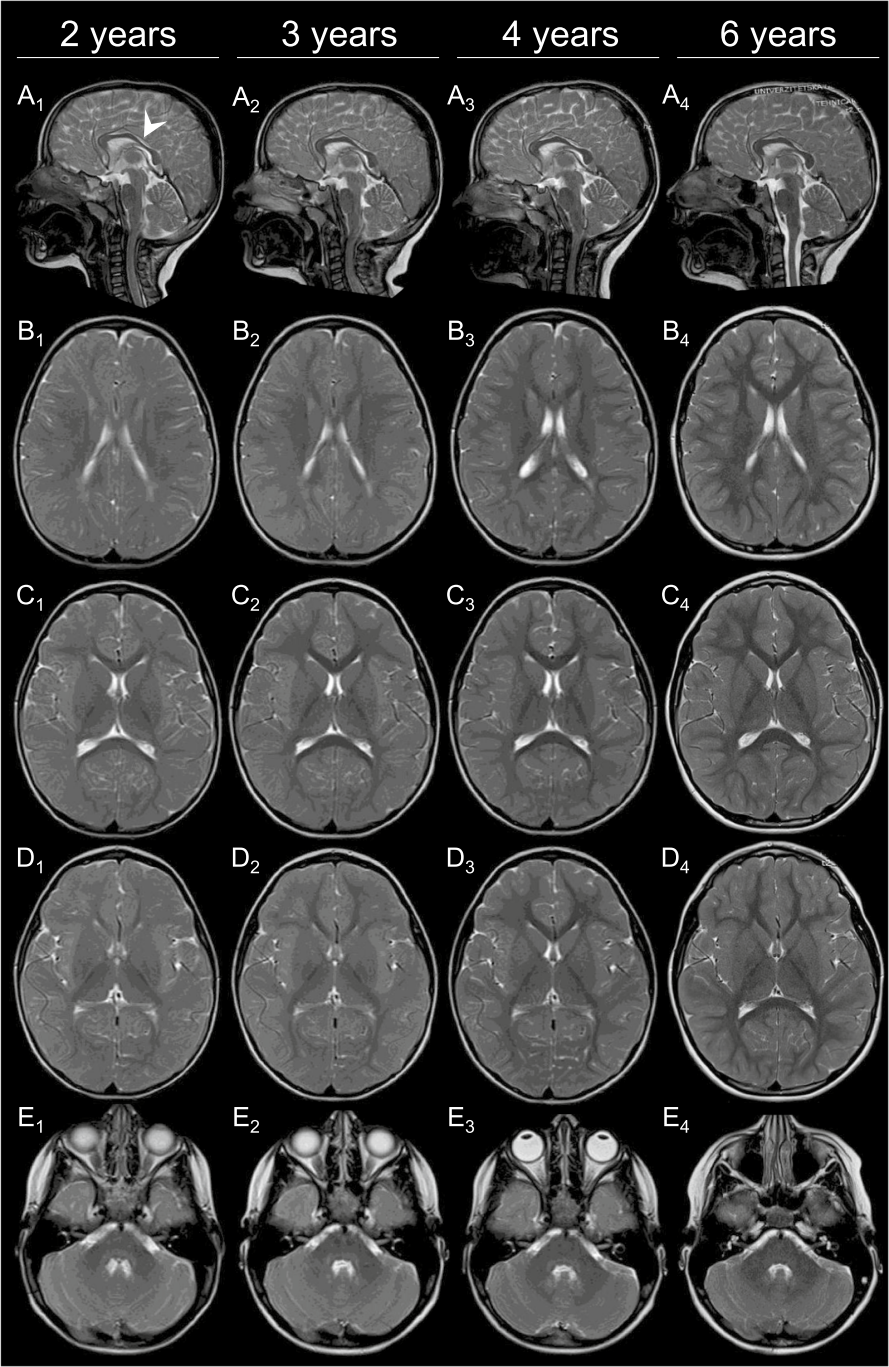
Brain MRI of Patient 2 from age 2 to 6 years. T2-weighted images are shown at 2 years (column 1: **A**_**1**_–**E**_**1**_), 3 years (column 2: **A**_**2**_–**E**_**2**_), 4 years (column 3: **A**_**3**_–**E**_**3**_) and 6 years (column 4: **A**_**4**_–**E**_**4**_). Sagittal (panel A) images show mild thinning of the corpus callosum (white arrowhead) but otherwise normal midline structures. In panels **B**, **C**, **D** and **E**, delayed myelination is appreciated, with complete myelination achieved only at age 6 years (**B**_**4**_, **C**_**4**_, **D**_**4**_ and **E**_**4**_)

In Patient 1, brain MRI at the age of 7 years (Fig. 1A3–E3) showed diffuse supratentorial hypomyelination, with relative preservation of myelination in the internal capsule, the splenium and body of the corpus callosum (Fig. 1B3, C3, D3), with thinning of the corpus callosum (Fig. 1A3). Cerebellar white matter was also hypomyelinated (Fig. 1E3). There was cerebral and marked cerebellar atrophy (Fig. 1A3–E3). When compared with MRIs obtained at age 5 years (Fig. 1A2–E2) and 2 years (Fig. 1A1–E1), the degree of hypomyelination was stable, but progression of cerebral and cerebellar atrophy was evident. These findings were consistent with a hypomyelinating leukodystrophy. In Patient 2, the first brain MRI at age 2 years revealed insufficient cerebral and cerebellar myelination for age, with a pattern similar to Patient 1, but with milder thinning of the corpus callosum and without cerebellar atrophy (Fig. 2A1–E1). Brain magnetic resonance spectroscopy (MRS) showed decreased white matter choline in both patients.

### Clinical laboratory measurements

For both patients, routine blood analyses and urinalyses, including blood/urine metabolic screening (lactate, pyruvate, amino-acids, organic acids, very-long-chain fatty acids) and vitamin B12 and homocysteine concentrations in serum, were normal. Of note, serum folate concentration values were also within normal range in both patients, measured at 17.8 ngr/ml in Patient 1, and 15.3 ngr/ml in Patient 2 (normal range: 3.1–20.5 ngr/ml).

Lumbar punctures to measure CSF neurotransmitters were not performed due to lack of parental approval and resources in Serbia. Therefore, the most efficient and the least invasive method to investigate the genetic diagnosis was to promptly perform exome sequencing using patient DNA extracted from whole blood.

### Genetic analysis

Using exome sequencing, in both siblings we identified a homozygous novel pathogenic variant in *FOLR1*: c.465_466delinsTG; p.W156G (NM_016725.3), which we assessed for pathogenicity using the ACMG guidelines and classifications. Using Sanger sequencing, we validated the presence of the variant in both patients and confirmed the parents to be heterozygous carriers (PP1). This specific variant has not been reported in large population databases (gnomAD; https://gnomad.broadinstitute.org/) (PM2), and a missense variant causing the same protein change is reported in heterozygous form in only 3 individuals, with no homozygous individuals reported (minor allele frequency = 0.00001061). This variant is reported by in silico softwares to be pathogenic and is present in a conserved amino acid region (PP3). Additionally, this specific indel variant has not been reported in the literature, however a missense variant at the same position leading to the same protein change (c.466 T > G; p.W156G) has been reported in two affected siblings in a compound heteozygous form [9], and one affected female in a homozygous form [10] (Table 1) (PS1). Therefore, the genetic diagnosis for both patients was confirmed, with the opportunity to treat this disease with folinic acid.

**Table 1:** Clinical, radiological, and genetic features, therapeutic protocols and treatment outcomes in patients with biallelic pathogenic variants in *FOLR1* reported in the literature, a (2009–2012), b (2013–2017), c (2019–2022)

Patient gender, ethnicity	Age at onset	1st Symptom	Motor signs	Seizures	Behavioral abnormality	*FOLR1* mutations	Pre-treatment Brain MRI	Brain MRS	Tx with folinic acid, *Age at Tx onset	Outcome, *Age at last follow-up	References
*a*											
M, German	2.5 y	Ataxia	Cerebellar, athetosis	+	+	c.352C > T (p.Q118*), c.525C > A (p.C175*)	Hypomyelination, cerebral and cerebellar atrophy, diffuse T2-hyperintensity of periventricular and subcortical WM	Low Cho and Ino	2–5 mg/kg/d oral *4y 7 m	Incomplete amelioration, *7y 7mo	Steinfeld et al. [1], Grapp et al. [2]
F, German	2y 3mo	Tremor, ataxia	Cerebellar	–	–	c.352C > T (p.Q118*), c.525C > A (p.C175*)	Cerebellar atrophy	N/A	5 mg/kg/d oral and 100 mg/week i.v. *2y 3mo	Complete clinical recovery, without radiological recovery, *5y 3mo	Steinfeld et al. [1], Grapp et al. [2]
F, Italian	2 y	Speech delay	Cerebellar, pyramidal	+	+	c.130_147dup (p.K44_P49dup) homozygous	Hypomyelination, cerebellar atrophy, focal T2-hyperintense WM lesions	N/A	2–5 mg/kg/d oral *5y	Incomplete amelioration, *8y	Steinfeld et al. [1], Grapp et al. [2]
M, Gambian	2 y	Ataxia, speech delay	Cerebellar, chorea	+	+	c.313 T > C (p.C105R) homozygous	Hypomyelination, cerebellar atrophy, focal T2-hyperintensities of periventricular WM	Low Cho	4 mg/kg/d oral *7y 8mo	Incomplete amelioration, *9y 8mo	Perez-Duenas et al. [20]
F, Finnish	1 y	Motor developmental delay	Cerebellar, athetosis, pyramidal	+	–	c.506G > A (p.C169Y) homozygous	Hypomyelination, cerebral and cerebellar atrophy, T2-hyperintense WM lesions	Low Cho	2–5 mg/kg/d oral *N/A	Incomplete amelioration, *N/A	Grapp et al. [2]
M, Azerbejani	22 mo	Seizures, developmental delay	Cerebellar	+	+	c.195C > G (p.C65W) homozygous	Delayed myelination, cerebellar atrophy, T2-hyperintensity of periventricular and subcortical WM	Normal	2–5 mg/kg/d oral *N/A	Incomplete amelioration, *N/A	Grapp et al. [2]
F, Finnish	3 mo	Microcephaly, tonus imbalance	Cerebellar, pyramidal	+	+	c.506G > A (p.C169Y) homozygous	Hypomyelination, cerebral and cerebellar atrophy	Normal	2–5 mg/kg/d oral *15y	Incomplete amelioration, *N/A	Grapp et al. [2]
2012											
F, Finnish	3 y	Nystagmus	Cerebellar, pyramidal	+	–	c.506G > A (p.C169Y) homozygous	Delayed myelination, cerebellar and cerebral atrophy, T2- hyperintensity in corticospinal tracts	N/A	2–5 mg/kg/d oral *14y	Incomplete amelioration, *N/A	Grapp et al. [2]
F, Finnish	2 y	Ataxia	Cerebellar	+	+	c.506G > A (p.C169Y), c.665A > G (p.N222S)	Irregular myelination, cerebellar atrophy	Low Cho and Ino	2–5 mg/kg/d oral *N/A	Incomplete amelioration, *N/A	Grapp et al. [2]
M, Finnish	1.5 y	Motor delay	Cerebellar, athetosis, pyramidal	+	+	c.506G > A (p.C169Y) homozygous and *POLG1* heterozygous mutations	Hypomyelination, T2-hyperintense WM lesions, cerebellar atrophy	N/A	2–5 mg/kg/d oral *N/A	No amelioration, *N/A	Grapp et al. [2]
F, Turkish	2 y	Global developmental delay	Cerebellar, pyramidal	+	+	g.3576 T > G homozygous, splice site variant	Hypomyelination, cerebral and cerebellar atrophy, thin corpus callosum	N/A	2–5 mg/kg/d oral *N/A	Incomplete amelioration, *N/A	Grapp et al. [2]
*b*											
M, Japanese	1 y	Ataxic gait	Cerebellar, pyramidal	+	_	c.374G > T (p.R125L), c.466 T > G (p.W156G)	Decreased cerebral WM volume with normal WM signal, cerebral subcortical calcifications, cerebellar atrophy, mild brainstem atrophy	N/A	Oral, dose N/A, *17y	N/A, *17y	Ohba et al. [9]
F, Japanese	2 y	Ataxic gait	Cerebellar	+	–	c.374G > T (p.R125L), c.466 T > G (p.W156G)	Cerebral subcortical calcifications, calcifications of basal ganglia, cerebellar atrophy	N/A	Oral, dose N/A, *14y	N/A, *14y	Ohba et al. [9]
M, Ghanaian	2.5 y	Hyperactive behaviour	Cerebellar, chorea, stimulus-responsive drop attacks	+	+	c.610C > T (p.R204*) homozygous	Cerebral hypomyelination, cerebellar atrophy, basal ganglia calcifications	Low Cho and Ino	5.6 mg/kg/d oral and 3 months trial with additional 120 mg/week i.v., *5y	Incomplete amelioration, *5.5y	Toelle et al. [21]
M, Saudi	2 y	Global developmental delay	Cerebellar, pyramidal	+	+	c.398C > A (p.P133H) homozygous	Cerebral hypomyelination, cerebellar atrophy	Normal	1.7 mg/kg/d oral, *5y 8mo	Incomplete amelioration, *7y 8mo	Al- Baradie et al. [19]
F, Saudi	2 y	Global developmental delay	Cerebellar, pyramidal	+	+	c.398C > A (p.P133H) homozygous	Cerebral hypomyelination, cerebellar atrophy	Normal	2 mg/kg/d oral, *4y	Incomplete amelioration, *6y	Al- Baradie et al. [19]
F, Italian	22 mo	Seizures	Cerebellar	+	-	c.128A > G (p.H43R) homozygous	N/A	N/A	2 mg/kg/d oral, *33y	Incomplete amelioration, *33y	Ferreira et al. [14]
F, Italian	18 mo	Ataxia	Cerebellar, bulbar	+	-	c.128A > G (p.H43R) homozygous	Cerebral hypomyelination, frontal cerebral atrophy, cerebellar atrophy	N/A	2 mg/kg/d oral, *28y	Incomplete amelioration, *28y	Ferreira et al. [14]
F, Belgian	3y 2mo	Global developmental delay	Cerebellar, chorea	+	+	c.332G > T (p.E108*), c.373C > T (p.R125C)	Hypomyelination	Low Cho	5 mg/kg/d oral, then 7 mg/kg/d oral and 20-25 mg/kg i.v. monthly, *5y	Incomplete amelioration, *7y	Delmelle et al. [12]
F, Belgian	2y 7mo	Global developmental delay	Cerebellar	+	_	c.332G > T (p.E108*), c.373C > T (p.R125C)	Hypomyelination	N/A	5 mg/kg/d oral, then 7 mg/kg/d oral and 20-25 mg/kg i.v. monthly, *3y 1mo	Incomplete amelioration, *5y 1mo	Delmelle et al. [12]
F, Turkish	3 y	Seizures	Dystonia, parkinsonism, cerebellar	+	–	c.383G > A (p.R128Q) homozygous	Basal ganglia calcification, cerebral atrophy,T1 hyperintensity in cerebral white matter	N/A	3 mg/kg/d oral for one month, then 5 mg/kg/d oral, *21y	IncompleteAmelioration, *22.5y	Karin et al. [16]
F, Russian	1.5 y	Seizures	Cerebellar, pyramidal	+	–	c.466 T > G (p.W156G) homozygous	Cerebral hypomyelination, cortical laminar necrosis and ulegyria in bilateral tempral lobes, cerebellar atrophy	N/A	2 mg/kg/d oral and 6 month trial 4 mg/kg/d i.m., then 2.5 mg/kg/d oral and 1 month trial 4 mg/kg/d i.v., *8y	Incomplete amelioration, *8y 7mo	Kobayashi et al. [10]
*c*											
F, Saudi	2 y	Tremor, speech delay	Intention tremor in upper limbs, hypotonia	–	–	c.665A > G (p.N222S) homozygous	T2W hyperintensities in cerebral subcortical and deep periventricular regions, centrum semiovale, more posteriorly	Normal	50 mg/d oral, *8y	Stable, *9.5y	Tabassumet al. [15]
M, Chinese	1.5 y	Global developmental delay	Cerebellar	+	+	c.524G > T (p.C175F) homozygous	Focal areas of cerebral encephalomalacia and laminar necrosis with diffuse cerebral white matter abnormality, cerebellar atrophy	N/A	2 mg/kg/d/ i.v. for one week and 6 mg/kg/d oral, then 11 mg/kg/d oral, *6y 11mo	Incomplete amelioration, *7y 7mo	Zhang et al. [17]
F, N/A	1.5 y	Ataxia, speech delay	Cerebellar	+	–	c.197G > A (p.C66Y) homozygous	Hypomyelination of infratentorial structures and cerebellum, cerebellar atrophy, calcifications of basal ganglia and subcortical white matter	N/A	8.9 mg/kg/d oral and 500 mg/week i.v., *11y	Incomplete amelioration, *11y 2mo	Mafi et al. [23]
F, Algerian	15 mo	Global developmental delay	Cerebellar, pyramidal, dysphagia	+	–	c.428G > A (p.W143*) homozygous	Diffuse supratentorial T2 hyperintensity, cerebellar atrophy	Low Cho and Ino	4.5 mg/kg/d oral and 300 mg/month i.v., initiated more than 15y after the onset, *17y	No amelioration, *18y	Brunetti et al. [18]
F, Algerian	10 mo	Unilateral strabismus, global developmental regression	Cerebellar, pyramidal, dystonia, dysphagia	+	–	c.428G > A (p.W143*)homozygous	Cerebellar atrophy with supratentorial fronto-parietal T2 hyperintensity	Low Cho and Ino	4.5 mg/kg/d oral and 300 mg/month i.v., initiated more than 14y after the onset, *15y	No amelioration, *16y	Brunetti et al. [18]
M, Algerian	9 mo	Unilateral strabismus, global developmental regression	Cerebellar, pyramidal, dystonia, dysphagia	+	_	c.428G > A (p.W143*) homozygous	Cerebellar atrophy and scattered T2 hyperintensity in supratentorial white matter	Low Cho and Ino	4.5 mg/kg/d oral and 300 mg/month i.v., initiated more than 13y after the onset, *14y	No amelioration, *15y	Brunetti et al. [18]
F, Turkish	1.5 y	Speech delay, autistic features	Cerebellar	+	+	c.665A > G (p.N222S) homozygous	Cerebral cortical atrophy	N/A	9 mg/kg/d oral and 24 mg/kg/month i.v. for 6 months, then 6 mg/kg/week i.v., *6y	Incomplete amelioration, *7.5y	Kanmaz et al. [22]
F, Greek	15 mo	Developmental delay	Cerebellar ataxia, hypotonia	infantile spasms, and other	+	c.195C > G (p.C65W), c.427 T > A (p.W143R)	Delayed myelination	Low Cho and Ino	3–6 mg/kg/d oral and 10 mg/kg i.v. twice weekly, *2.5y	Incomplete amelioration, *3.5y	Papadopoulou et al. [13]
M, Greek	12 mo	Developmental stagnation and delay, dyskinesia	Hypotonia, dyskinesia	–	+	c.195C > G (p.C65W), c.427 T > A (p.W143R)	Delayed myelination	Low Cho and Ino	2–6 mg/kg/d oral, *12 m	Incomplete amelioration, *2y	Papadopoulou et al. [13]
M, Serbian	1.5 y	Gait ataxia, speech delay	Cerebellar, bulbar, pyramidal	+	+	c.465_466delinsTG (p.W156G) homozygous	Supratentorial hypomyelination, affected corpus callosum, cerebellar hypomyelination and atrophy	Low Cho	2–5 mg/kg/d oral for 8 m, then 8 mg/kg/d oral, *8y	Incomplete amelioration, *12y	Current report Patient 1
F, Serbian	22 mo	Intention tremor in upper limbs	Intention tremor in upper limbs, truncal ataxia	–	–	c.465_466delinsTG (p.W156G) homozygous	Cerebral and cerebellar hypomyelination, thin corpus callosum	Low Cho	2–5 mg/kg/d for 12 m, then 7 mg/kg/d oral, *24mo	Complete clinical and radiological Recovery, *6y	Current report Patient 2

M = male; F = female; y = years; mo = months; MRI = magnetic resonance imaging, MRS = pre-treatment magnetic resonance spectroscopy; Cho = choline;Ino = inositol; i.v. = intravenously; N/A = not assessed, Tx = therapy

### Treatment and response to therapy

Treatment with folinic acid was initiated immediately after obtaining the genetic results, (i.e., at 8 years of age in Patient 1 and at 2 years of age in Patient 2) and the response to therapy was monitored over 4 years. Specifically, the patients were treated with levofolinic acid, the L-isomer of folinic acid. Notably, folinic acid can also be prescribed as a mixture of both the biologically active L-isomer and the inactive D-isomer, however, reports show that a better outcome may be associated with the use of only the L-folinic acid compound [11].

In Patient 1, the initial dose of oral folinic acid of 2 mg/kg/day did not lead to notable improvements, and therefore within a month, the dose was increased to 5 mg/kg/day, which resulted in a dramatic improvement of neurological features. The patient’s bulbar symptoms disappeared, weakness and ataxia began to subside, and over the next 8 months, he gradually began to walk independently, while his speech comprised 4–5 meaningful words. His dose of oral folinic acid was then slowly increased to 8 mg/kg/day. The severity and frequency of seizures decreased from dozens per day to 0–3 brief atonic and focal seizures with impaired awareness, and the antiepileptic therapy was reduced to two AEDs which are presumed to have a minimal anti-folate effect (levetiracetam and lamotrigine). Any attempt to further modify/reduce antiepileptic therapy would result in the aggravation of seizures. During folinic acid treatment, his serum folate concentration remained within the normal range, with values measured at 17.1 ngr/ml (normal 3.1–20.5 ngr/ml) at 12 years of age.

On the latest neurological examination at age 12 years, the patient presented with cerebellar signs, while bulbar and pyramidal signs were completely resolved. Cerebellar ataxia and hypotonia appeared milder, and he could walk and perform simple motor tasks independently. His behavioral abnormalities subsided, however, no significant improvement in expressive language was observed. Follow-up brain MRI at 10 years of age showed progression of both supra- and infratentorial myelination (Fig. 1A4–E4), with a further improved myelination on the latest MRI at age 12 years (Fig. 1A5–E5). Brain MRS also improved, with normalization of the white matter choline peaks for age.

In Patient 2, neurological signs completely resolved after 3 months of treatment with 2 mg/kg/day of oral folinic acid. The patient has since been symptom-free and developing normally. Follow-up brain MRI at 3 years of age showed amelioration of the abnormal cerebral and cerebellar white matter signal, but without complete normalization of myelination (Fig. 2A2–E2). Folinic acid oral dose was then gradually increased to 7 mg/kg/day. Brain MRI at 4 years of age showed further improvement (Fig. 2A3–E3), and at 6 years of age myelination appeared normal (Fig. 2A4–E4). Her latest neurological examination at 6 years of age was normal. Her levels of serum folate also remained within the normal range, with the latest value at 6 years of age measuring 15.8 ngr/ml (normal range: 3,1–20.5 ngr/ml).

### Literature review

Our review of the pre-treatment clinical and brain MRI findings among 31 reported *FOLR1*-related patients (Table 1) revealed no notable genotype–phenotype correlations.

The age of the disease onset among the reported patients ranged from 3 months [2] to 3 years and 2 months [12], but in most cases, onset was between 1 year and 2.5 years of life. The commencement of folinic acid treatment ranged from ages 12 months [13] to 33 years [14]. Likewise, the time interval between onset of symptoms and folinic acid treatment initiation among patients ranged from almost immediately in two patients [1, 13] to a delay of more than 31 years in the oldest reported patient [14]. On average, the delay in therapy was 2–10 years.

The earliest reported symptoms were psychomotor regression and cerebellar ataxia. Epileptic seizures usually appeared afterwards, rarely before 18 months of age, and were of different types. They were not documented in three reported patients [1, 13, 15], while all the other patients manifested various combinations of myoclonic, atonic, tonic–clonic, tonic, absence seizures, epileptic spasms [10, 13], and/or focal seizures with and without impaired awareness. The most common were myoclonic seizures, observed in all but four patients [2, 13, 16, 17]. The seizures were commonly described as drug-resistant, of high frequency, and frequently evolving to status epilepticus.

Cerebellar signs were described in all patients and were typically accompanied by other neurological signs. Extrapyramidal motor signs were also present in 10 patients [1, 2, 12, 13, 16, 18, 20, 21]. Four patients had accompanied bulbar signs [14, 18], and 13 patients had accompanied pyramidal signs [1, 2, 9, 10, 18, 19]. Autistic behavioral features were observed in 18 patients [1, 2, 12, 13, 17, 19–22]. Congenital microcephaly was described in one patient [2], while acquired microcephaly was noted in five patients [2, 17, 21]. Head circumference was normal in all other patients.

The majority of patients had supratentorial hypomyelination of various degrees, with or without cerebellar atrophy [1, 2, 10, 12, 14, 18–21]. Cerebellar atrophy was absent in seven patients [12, 13, 15, 16, 22]. Four patients also had basal ganglia calcifications [9, 16, 21, 23], one patient had accompanied bilateral temporal cortical laminar necrosis and ulegyria [10], while one other patient had white matter encephalomalacia [17]. Two patients had no myelin abnormalities but cerebellar atrophy with or without cerebral calcifications [2, 9], and one had cerebral cortical atrophy only [22]. Delayed myelination with or without cerebellar atrophy was seen in four patients [2, 13]. Apart from the two patients described in this study, infratentorial hypomyelination was described only in one patient [23], while thinning of the corpus callosum was reported in one other [2]. MRS values before treatment showed low white matter choline and/or inositol in all patients except for five, which were normal [2, 15, 19].

The effect of folinic acid treatment has been associated with various clinical and radiological outcomes (Table 1). Regarding folinic acid administration, the recommendation is to give 2–10 mg/kg/day orally, with the suggestion to change the route of administration to intravenous or intrathecal if the response is suboptimal [24]. However, the dose of folinic acid and route of administration vary in different reports from 1.7 mg/kg/day orally [19] to the combination of 8.9 mg/kg/day orally with 500 mg/week/intravenously [23] (Table 1). Incomplete amelioration was accomplished in all but four patients [2, 18], regardless of the route of folinic acid administration. It should be noted that the lack of response to treatment in these patients was suggested to result from *POLG1* mutations additionally found in one patient [2], and a long delay in diagnosis (13–15 years) in the other three patients [18]. Interestingly, in the two oldest reported patients who had a delay in diagnosis of 27 years and 31 years respectively, administration of oral folinic acid at 2 mg/kg/day resulted in a marked reduction in the frequency of seizures, permitting a reduction of antiepileptic therapy and improvement of quality of life [14]. The best treatment results were observed in children who were diagnosed and treated early [1, 2, 12]. In addition to Patient 2 from the current study, complete clinical recovery was only accomplished in one other patient for whom folinic acid therapy was started immediately after the symptom onset in the second year of life [1]. Complete recovery of both clinical and radiological features, such as seen in Patient 2 of this study, has never been documented.

## Discussion

The siblings we describe here provide strong support for the effectiveness and importance of folinic acid treatment initiation at a very early age in patients with pathogenic variants in *FOLR1* and neurodegeneration due to cerebral folate deficiency. The younger sibling (Patient 2) is the first reported patient with neurodegeneration due to cerebral folate deficiency who demonstrated complete recovery of both clinical features and brain MRI abnormalities following oral folinic acid treatment started just after symptom onset.

Contrarily, the 6-year delay in diagnosis of Patient 1 can explain his incomplete clinical recovery. It is important to note that the main cause for the delay in diagnosis was the inability to provide timely access to metabolic and genetic testing, as the patients were treated in a department without the necessary resources to perform this testing on a clinical basis. Therefore, genetic analyses were only performed later on a research basis, and by the time of genetic diagnosis, his neurological impairments had more substantially progressed. In Patient 2, genetic analysis was performed near the beginning of symptom onset at 2 years of age, and the immediate initiation of folinic acid therapy led to the complete resolution of both clinical and MRI abnormalities.

Additionally, the prolonged exposure of Patient 1 to a plethora of antiepileptic drugs for intractable epilepsy prior to establishing the correct diagnosis may have resulted in negative effects on his disease course. Indeed, some AEDs may have a harmful anti-folate effect, including valproate, phenobarbital, primidone, phenytoin, carbamazepine, oxcarbazepine, topiramate, gabapentin, and pregabalin [25, 26]. However, specific AEDs such as lamotrigine, levetiracetam, clobazam, and clonazepam have not demonstrated notable interactions with cerebral folates [26].

Based on the published literature, psychomotor regression with cerebellar ataxia starting in the second to third year of life, along with refractory epilepsy with mostly myoclonic seizures and radiological findings of cerebral hypomyelination with or without cerebellar atrophy should raise suspicion of this disease. Other clinical signs such as autistic behavioral features, deceleration of head growth, frequent occurrence of status epilepticus, and radiological findings of brain calcifications can be seen as well and do not exclude the possibility of this disorder/diagnosis. Furthermore, due to the phenotypic variability associated with this disease and other inherited neurological diseases, all individuals with drug-resistant epilepsy should undergo genetic testing.

Prior to our report, a complete resolution of neurologic symptoms was only accomplished in one patient, however, their MRI features did not fully resolve, with the preexisting cerebellar atrophy remaining despite the therapy [1, 2]. This patient was treated with both oral and intravenous folinic acid, including 5 mg/kg/day orally and 100 mg/week intravenously [2].

Brain MRI features improved in both of our patients with oral folinic acid therapy. Specifically, white matter T2-signal hyperintensity started to decrease and the volume of both supra- and infratentorial structures began to increase after treatment for two years in Patient 1, and one year in Patient 2. After four years of treatment, the brain MRI findings in Patient 1 did not yet normalize, but continued to improve. Moreover, brain MRS revealed normalization of the white matter choline peaks for age, in line with the reactivation of the myelination process [27]. The 5-year evolution of Patient 1’s brain MRI features prior to treatment is the longest reported for this disease, showing progressive cerebral and cerebellar atrophy together with a pattern of hypomyelination. In Patient 2, the level of myelination completely recovered after four years of treatment, demonstrating the first complete radiological recovery from this disease.

The route of folinic acid administration may be of importance when treating neurodegeneration due to cerebral folate deficiency, given that FOLRα is necessary for the transfer of MTHF from the CSF to the brain parenchyma [2, 3]. In the absence of FOLRα, other transporters such as the reduced-folate-carrier and proton-coupled-transporter may transport MTHF across the blood–brain barrier, but due to their very low MTHF affinity, there is a need for high plasma MTHF concentrations (i.e., high folinic acid doses) [2, 3]. However, the further delivery of exosomes with MTHF from CSF into the brain parenchyma likely exclusively depends on FOLRα, which could explain the lack of complete recovery despite the application of high doses of folinic acid and despite the normalization of MTHF concentration in CSF [2, 3, 12]. Since even high doses of folinic acid are typically unable to fully overcome the lack of FOLRα, new therapeutic strategies are needed. These may include the application of FOLRα + exosomes into the CSF as proposed by Grapp et al. [3]. With recent advances in gene therapy development, this avenue is certainly also very appealing.

Although both siblings harbored the same homozygous pathogenic variant in *FOLR1*, they exhibited phenotypic variability as the older sibling demonstrated an earlier disease onset with a more severe disease course. It has been proposed that even in the presence of the same *FOLR1* pathogenic variants, the variable phenotypic severity may reflect the individual variability in the timing of fetal FOLRβ inactivation, different potency of FOLRβ functioning, variable residual FOLRα functions, or variable capacity of alternative folate transport mechanisms [2]. It may also reflect the different hypothesized processes through which folates contribute to myelin formation [2, 5]. Peripheral nerves were intact in our patients, while peripheral neuropathy was found in three reported patients, further suggesting a link between FOLRα and Schwann cells homeostasis [2, 10]. Regardless of the specific pathogenic mechanisms, folinic acid is the only disease-modifying therapy for the clinical and radiological manifestations of cerebral folate deficiency, and these cases demonstrate the importance of early treatment for the amelioration of disease features.

## Conclusions

We report a novel pathogenic variant in *FOLR1* in two Serbian siblings with clinical and brain MRI presentations consistent with neurodegeneration due to cerebral folate transport deficiency, along with the response to treatment and long-term follow-up, therefore contributing to the literature delineating the natural history of the disease. The youngest sibling is the first patient reported for whom complete recovery of both clinical and brain radiological abnormalities was achieved with oral folinic acid treatment, suggesting that early oral therapy may be sufficient to treat this condition compared to other more invasive routes of administration.

Furthermore, in patients with genetically undiagnosed hypomyelination, *FOLR1* should be investigated promptly and included in all leukodystrophy panels to ensure early treatment with folinic acid and optimize clinical outcomes. Finally, these cases highlight the importance of universal access to genetic testing, to ensure that treatable conditions are promptly diagnosed and treatment initiated early to optimize clinical outcomes.

## Data Availability

Anonymized data supporting the findings of this study not published within this article will be made available from the corresponding author, upon reasonable request.

## Abbreviations

*FOLR1*: Folate receptor-alpha gene
FOLRα: Folate receptor-alpha
FOLRβ: Folate receptor-beta
MTHF: 5-Methyltetrahydrofolate
CSF: Cerebrospinal fluid
MRI: Magnetic resonance imaging
MRS: Magnetic resonance spectroscopy
AED: Antiepileptic drug
